# Hyponatremia, hypernatremia and impairment of functional, psychological and sexual domains

**DOI:** 10.1007/s40618-023-02218-w

**Published:** 2023-10-26

**Authors:** D. Norello, G. Rastrelli, L. Antonio, G. Bartfai, F. F. Casanueva, A. Giwercman, I. T. Huhtaniemi, T. W. O’Neill, M. Punab, J. Slowikowska-Hilczer, J. Tournoy, D. Vanderschueren, F. C. W. Wu, M. Maggi, A. Peri

**Affiliations:** 1 Department of Experimental and Clinical Biomedical Sciences “Mario Serio”, University of Florence, Careggi Hospital, Viale Pieraccini, 6, 50139 Florence, Italy; 2 Department of Chronic Diseases and Metabolism, Laboratory of Clinical and Experimental Endocrinology, KU Leuven, Leuven, Belgium; 3 Department of Endocrinology, University Hospitals Leuven, Leuven, Belgium; 4 Department of Obstetrics, Gynaecology and Andrology, Albert Szent-Györgyi Medical School, University of Szeged, Szeged, Hungary; 5 Department of Medicine, CIBER de Fisiopatologıa Obesidad y Nutricion, Santiago de Compostela University, Complejo Hospitalario Universitario de Santiago (CHUS), Santiago de Compostela, Spain; 6 Department of Translational Medicine, Lund University, Malmö, Sweden; 7 Department of Metabolism, Digestion and Reproduction, Institute of Reproductive and Developmental Biology, Imperial College London, London, UK; 8 Centre for Epidemiology Versus Arthritis, The University of Manchester and NIHR Manchester Biomedical Research Centre, Manchester University NHS Foundation Trust, Manchester, UK; 9 Andrology Clinic, Tartu University Hospital, and Institute of Clinical Medicine, and Institute of Biomedicine and Translational Medicine, University of Tartu, Tartu, Estonia; 10 Department of Andrology and Reproductive Endocrinology, Medical University of Łódź, Łódź, Poland; 11 Department of Geriatrics, University Hospitals Leuven, and Department of Public Health and Primary Care, KU Leuven, Leuven, Belgium; 12 Department of Endocrinology, Manchester University NHS Foundation Trust, Manchester, UK

**Keywords:** Hyponatremia, Hypernatremia, Physical function, Psychologic function, Sexual function, Erectile dysfunction

## Abstract

**Objective:**

To determine the influence of serum sodium on physical, psychologic and sexual function.

**Methods:**

This is a cross-sectional survey on 3340 community-dwelling men aged 40–79 years from a prospective cohort study in eight European countries, the European Male Ageing Study (EMAS). Participants filled-out the Short Form-36 (SF-36), the Physical Activity Scale for the Elderly (PASE), and the EMAS sexual function questionnaire. For all the analyses, serum sodium corrected for glycaemia ([Na^+^]_G_) was used.

**Results:**

The relationship between [Na^+^]_G_ and SF-36 physical function score (F = 3.99; p = 0.01), SF-36 mental health score (F = 7.69; p < 0.001), and PASE score (F = 14.95; p < 0.001) were best described by a quadratic equation, with worse scores for [Na^+^]_G_ in either the lowest or the highest ends of the range. After dividing the sample into [Na^+^]_G_ < 136 mmol/L (n = 81), 136–147 mmol/L (n = 3223) and > 147 mmol/L (n = 36), linear regression analyses with linear spline functions adjusted for confounders did not confirm these relationships. Similarly, erectile dysfunction and [Na^+^]_G_, were in a quadratic relationship (F = 9.00; p < 0.001). After adjusting for confounders, the linear regression with spline functions denoted a significantly worsened erectile function for increases in serum [Na^+^]_G_ > 147 mmol/L (B = 0.15 [0.04;0.26], p < 0.01) but no relationship with [Na^+^]_G_ < 136 mmol/L. Likewise, the relationship of [Na^+^]_G_ with concerns about sexual dysfunction was confirmed only for men with serum [Na^+^]_G_ > 147 mmol/L.

**Conclusions:**

This is the first study supporting an association between [Na^+^]_G_ and sexual function. A worsening of erection and concerns about sexual function were observed for the highest values of [Na^+^]_G_, independently of other relevant factors.

## Introduction

Hyponatremia is the most common electrolyte disorder in clinical practice and mild forms occur in up to 30% of hospitalized patients [1, 2]. The Syndrome of Inappropriate Antidiuresis (SIAD) is frequently involved, though there are many other endocrine and non-endocrine causes [3].

Neurological manifestations of symptomatic hyponatremia depend on the entity of hyponatremia, but even more on its rapidity of onset. Symptomatic patients with limited manifestations, usually due to chronic hyponatremia, can show clinical features such as headache, irritability, nausea, vomit, mental slowdown, confusion, delirium, disorientation, and equilibrium alterations. Patients with acute hyponatremia often present with more severe symptoms, and life threatening manifestations such as stupor, coma, seizures, and respiratory arrest may occur [4, 5].

Hypernatremia is characterized by the presence of a deficit of water in relation to the body’s sodium stores, which can be determined by a net water loss (pure water loss, including diabetes insipidus, or hypotonic fluid loss) or a hypertonic sodium gain [6]. As with hyponatremia, clinical features depend on the level and rapidity of increase of serum sodium [7]. Patients can show irritability, nausea, abdominal pain, muscle weakness, lethargy, confusion, convulsions and ultimately coma. In the brain, hypernatremia may result in cerebral bleeding, subarachnoid hemorrhage, and permanent neurologic damage or death [8–10]. Hypernatremia is associated with an increased risk of mortality, though it is difficult to tease out the contributions of the hypernatremia per se and the underlying diseases [11].

While the clinical manifestations of hyponatremia and hypernatremia are well defined for severely altered serum sodium values, less is known about minor changes in serum sodium. There is some evidence that mild reductions in serum sodium are associated with adverse health consequences including an increased risk of falls, bone fractures [12], impairment of neurocognitive and motor performance, mood disorders [13], and an increased risk of mortality [14]. However, there is a relative paucity of data.

The European Male Ageing Study (EMAS) is a cohort of community-dwelling men aged 40–79 years [15–17]. Using data from the study, the aim of this analysis is to determine the influence of serum sodium on physical and psychologic health and quality of life, including sexual function. In particular, we aim at evaluating the influence of serum sodium on these parameters for values at the extreme ends of the range of this population.

## Materials and methods

### Design

Subjects were recruited for participation in the EMAS study, a population-based study of ageing in eight European centers [15–17]. Details about recruitment, response rates, and assessments have been described elsewhere [15].

In brief, men aged 40–79 years were recruited from population-based registers in eight European centers: Florence (Italy), Leuven (Belgium), Łódz´ (Poland), Malmö (Sweden), Manchester (UK), Santiago de Compostela (Spain), Szeged (Hungary), and Tartu (Estonia). Stratified random sampling was used with the aim of recruiting equal numbers of men into each of the following age bands: 40–49, 50–59, 60–69, and 70–79 years. Participants were contacted by letter, asked to complete a short postal questionnaire and invited to attend for assessment at a local health centre/clinic. Overall, the mean response rate for full participation in the study was 41%. After a mean of 4.3 years, subjects enrolled were invited to take part in follow-up survey with similar assessments. For the specific purpose of this study, only baseline data were considered. Ethical approval was obtained in each center.

### Questionnaires and physical measures

The postal questionnaire included information about self-reported health, concomitant morbidities, employment, education, smoking, and alcohol consumption [15]. Those who agreed to attend for baseline assessment were asked to complete an interviewer-assisted series of questionnaires, had a number of performance/functional assessments, measurement of blood pressure, height, weight, waist circumference, and a fasting blood sampling.

Among the questionnaires provided, the Short Form-36 (SF-36) [18], the Physical Activity Scale for the Elderly (PASE) [19], and the EMAS sexual function questionnaire (EMAS-SFQ) [20] has been filled out by the participants.

The SF-36 was designed for use in clinical practice and research, health policy evaluations, and general population surveys, and explores quality of life, including mental and physical health, through one multi-item scale [18].

The PASE is a brief, easily scored, reliable and valid instrument for the assessment of physical activity [19].

The EMAS-SFQ is a validated 16-item questionnaire exploring sexual function specifically developed for the EMAS [20]. It explores sexual functioning, sexual function-related distress, and change in sexual functioning compared with 1 year ago and showed excellent reliability and validity [17, 20, 21].

### Blood samples

A single fasting morning (before 10:00 am) venous blood sample was obtained and processed serum was stored at −80°C.

Common and standardized measurements, including full blood count, lipids, glucose, and serum sodium, were performed in each centre.

Analyses for high-density lipoprotein (HDL) cholesterol and triglycerides were performed using commercially available enzymatic methods. Fasting serum glucose was measured using standard hexokinase enzymatic assays. Serum sodium was assayed with ion selective electrode method.

Measurement of prolactin and thyroid-stimulating hormone was performed using a chemiluminescence immunoassays. Testosterone was assayed by gas chromatography-mass spectrometry [22]. All clinical pathology laboratories were accredited by the relevant national authorities and observed current guidelines on Good Laboratory Practice (GLP).

### Metabolic syndrome

The metabolic Syndrome was defined using the American Heart Association (AHA) criteria with the presence of three of these five conditions: waist circumference ≥ 102 cm, triglycerides ≥ 150 mg/dL or on hypertriglyceridemia treatment, HDL cholesterol < 40 mg/dL (men) or on treatment for reduced HDL cholesterol, systolic blood pressure ≥ 130 mm Hg and/or diastolic blood pressure ≥ 85 mm Hg or on antihypertensive treatment, fasting glucose ≥ 100 mg/dL or on hyperglycemia treatment [23].

### Statistical analysis

Descriptive statistics were used to characterize the study population.

In order to account for the impact of glycaemia on serum sodium concentration, the values of sodium corrected for serum glucose (mmol/L) were used, according to a previously published formula [24, 25]:

Serum sodium + [(serum glucose/0.0555—100) × 0.016] if serum glucose is ≤ 22.2 mmol/L;

Serum sodium + [(serum glucose/0.0555—100) × 0.04] if serum glucose is > 22.2 mmol/L.

From this point onwards, when using serum [Na^+^]_G_ we will refer to serum sodium corrected for glycaemia.

Firstly, we assessed the best relationship describing the association between serum [Na^+^]_G_, considered as a continuous variable, and the different questionnaire domains by fitting different curves. Afterwards, we divided the sample into three groups according to thresholds of serum [Na^+^]_G_, as derived from the observation of the best fitting curve (i.e. quadratic) for the relationship between serum [Na^+^]_G_ corrected for glucose and questionnaire scores (i.e. < 136 mmol/L, 136–147 mmol/L and > 147 mmol/L). Then, we performed linear regression analyses with linear spline functions set at the aforementioned thresholds adjusted for EMAS centre, age, morbidities, medications, body mass index (BMI), and total testosterone to explore the relationship between serum [Na^+^]_G_ and the questionnaire scores.

In order to account for multiple comparisons, the Bonferroni adjustment was applied.

Statistical analyses were performed using software IBM SPSS version 28 and software STATA Statistics Data Analysis version 13.1.

## Results

### Participants

Of 3369 participants, 23 with missing serum sodium data and 6 with missing glycaemia data were excluded from the analysis. The characteristics of the remaining 3340 men are shown in Table 1.

**Table 1:** Characteristics of the study population (n = 3340)

	Mean	SD
Age (years)	59.97	10.99
Serum sodium (mmol/L)	140.97	2.56
Serum sodium corrected for glycaemia (mmol/L)	141.01	2.53
Prolactin (ng/mL)	8.57	6.52
Thyroid stimulating hormone (mU/L)	1.72	2.26
Total testosterone (nmol/L)	16.44	5.86
Body mass index (kg/m^2^)	27.67	4.07
Waist circumference (cm)	98.47	11.01
Hip circumference (cm)	99.81	7.62
Waist: Hip ratio	0.98	0.06
Total cholesterol (mmol/L)	5.54	1.24
HDL cholesterol (mmol/L)	1.40	0.37
Triglycerides (mmol/L)	1.57	1.16
Glucose (mmol/L)	5.64	1.38
SF-36 physical function score	27.12	3.82
SF-36 mental health score	20.32	3.47
EMAS-SFQ erection ability	2.05	1.03
EMAS-SFQ erection worry	1.54	0.91
EMAS-SFQ frequency worry	1.46	0.83
EMAS-SFQ orgasm worry	1.50	0.85
EMAS-SFQ morning erection worry	1.19	0.60
PASE	195.61	91.77

	Number	%
Metabolic syndrome (AHA)	1276	38.2
Waist circumference > 102 cm	1115	33.4
Blood pressure ≥ 130/85 mmHg†	2781	83.3
HDL cholesterol < 1.03 mmol/L	787	23.6
Triglycerides ≥ 1.7 mmol/L	1292	38.7
Glucose ≥ 5.6 mmol/L‡	1411	42.2
Diabetes§	248	7.4
Current smoker	700	21.0
Drugs (≥ 1)	1830	54.8
Anti-psychotic drug use	17	0.5
Anti-depressant drug use	122	3.7
Morbidities (≥ 1)	2275	68.1
Cardiovascular diseases	1204	36.0
Pituitary diseases	13	0.4

The specific type of morbidities and drugs investigated were previously reported elsewhere^15^

*HDL* high-density lipoprotein, *SF36* the short form-36^18^, *EMAS-SFQ* European Male Aging Study sexual function questionnaire^20^, *PASE* Physical Activity Scale for the Elderly^19^, *SD* standard deviation, *AHA* American Heart Association

^†^Measured blood pressure and/or usage of anti-hypertensive drugs

^‡^Measured blood glucose and/or usage of antidiabetic drugs

^§^Self-report and/or usage of antidiabetic drugs

Mean age was 59.97 years (SD = 10.99), mean BMI was 27.67 kg/m^2^ (SD = 4.07), 21% were current smokers, while the mean serum sodium was 140.97 mmol/L (SD = 2.56) and the mean serum [Na^+^]_G_ was 141.01 mmol/L (SD = 2.53). Serum [Na^+^]_G_ concentrations were not significantly associated with the age of the participants, alcohol intake, smoking habits or BMI (B = 0.01 [− 0.01; 0.01], p = 0.10; B = − 0.03 [− 0.09; 0.02], p = 0.19; B = − 0.02 [− 0.23; 0.19], p = 0.85, and B = − 0.05 [− 0.10; 0.01], p = 0.08, respectively).

Concerning the comorbid conditions, 7.4% of the patients were diabetic, 83.3% had blood pressure ≥ 130/85 mmHg and/or used of anti-hypertensive drugs, 36.0% reported history of cardiovascular diseases, and 38.2% fulfilled the AHA criteria for metabolic syndrome [23].

Furthermore, 54.8% of the participants reported to take at least one medication, in particular 4% took an anti-depressant or anti-psychotic drug.

### Correlations between serum [Na^+^]_G_, mental health, physical domains, and sexual function

To evaluate the possible associations between serum [Na^+^]_G_, considered as a continuous variable, and mental and physical health self-perception or physical activity, we analyzed the relationships with SF-36 and PASE questionnaires.

We began by observing the relationship that best describes the association with serum [Na^+^]_G_ and the different questionnaire domains.

**Fig. 1:**
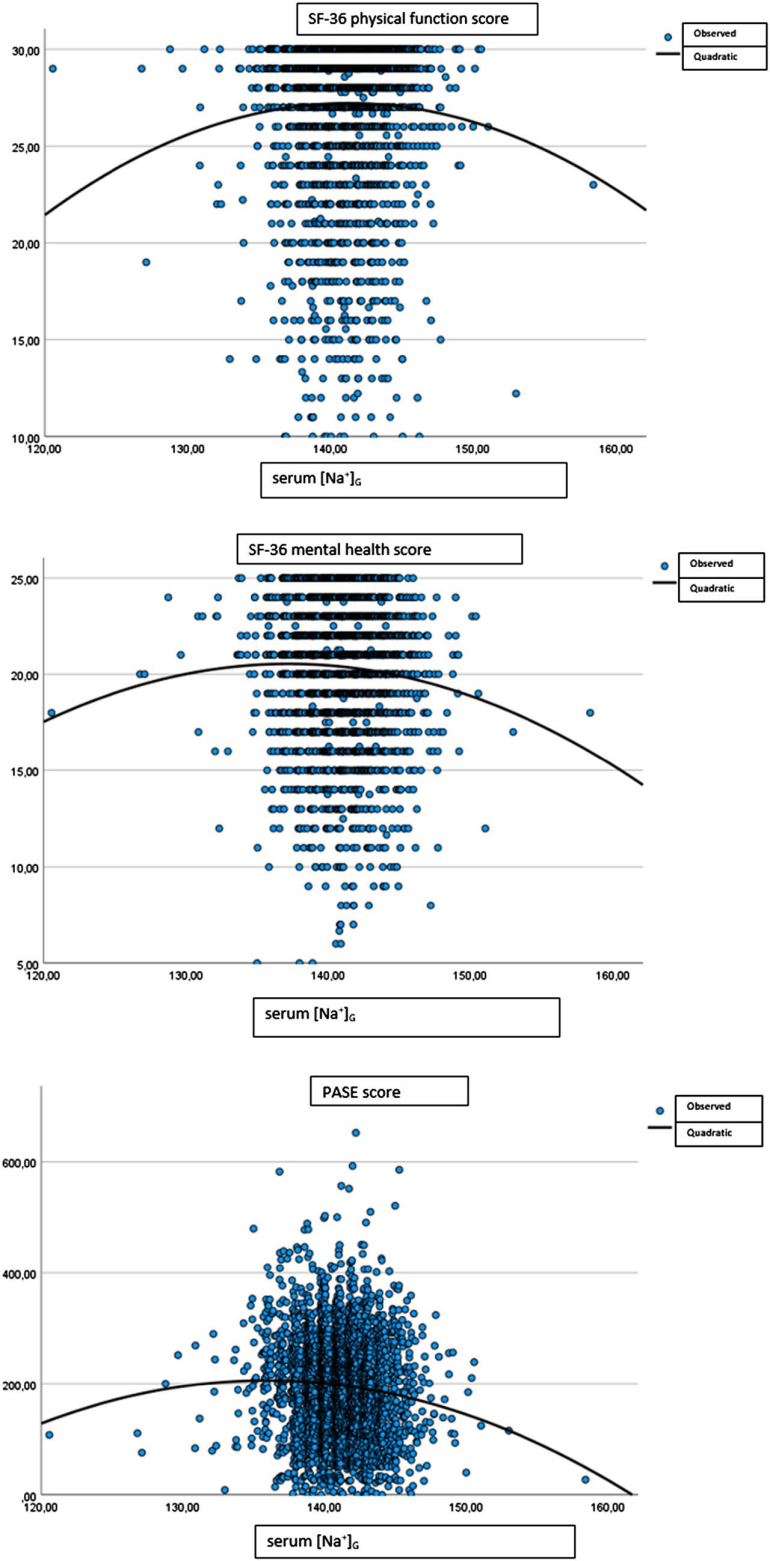
Relationship between serum [Na^+^]_G_, mental health and physical domains

We found that the relationship between serum [Na^+^]_G_ and SF-36 physical function score (F = 3.99, p = 0.01), SF-36 mental health score (F = 7.69, p < 0.001), and PASE score (F = 14.95, p < 0.001) were best described by a quadratic equation. According to the quadratic relationship, a worsening in the questionnaire scores was observed for values of serum [Na^+^]_G_ in either the lower or the higher ends of the range (Fig. 1).

By visually inspecting the quadratic curves, we identified possible threshold values for serum [Na^+^]_G_ around 136 mmol/L and 147 mmol/L. Therefore, we divided the sample into three groups according to serum [Na^+^]_G_ (< 136 mmol/L, n = 81; 136–147 mmol/L, n = 3223; and > 147 mmol/L, n = 36) and we performed linear regression analyses with linear spline functions for the relationship between serum [Na^+^]_G_ and the questionnaire scores adjusted for centre, age, morbidities, medications, BMI, and total testosterone. We did not find any significant relationship for SF-36 physical function score, SF-36 mental health score or PASE score (Table 2).

**Table 2:** Linear regression analysis with spline functions for the relationship between serum [Na^+^]_G_ adjusted for EMAS centre, age, morbidities, drugs, BMI, total testosterone

	Questionnaire score (mean ± SD)	B	95% confidence interval	p value	B change	95% confidence interval	p value
SF-36 physical function score							
[Na^+^]_G_ < 136 mmol/L	27.02 ± 3.80	0.04	− 0.21; 0.30	0.72	–	–	–
136 ≤ [Na^+^]_G_ ≤ 147 mmol/L	27.14 ± 3.82	0.06	0.01; 0.12	0.03	0.01	− 0.25; 0.28	0.91
[Na^+^]_G_ > 147 mmol/L	26.08 ± 4.34	− 0.31	− 0.71; 0.10	0.13	− 0.35	− 0.76; 0.07	0.10
SF-36 mental health score							
[Na^+^]_G_ < 136 mmol/L	20.71 ± 3.74	0.08	− 0.65; 0.82	0.82	–	–	–
136 ≤ [Na^+^]_G_ ≤ 147 mmol/L	20.33 ± 3.46	0.07	− 0.08; 0.23	0.34	0.05	− 0.21; 0.31	0.70
[Na^+^]_G_ > 147 mmol/L	18.69 ± 3.64	− 0.25	− 1.38; 0.87	0.65	− 0.10	− 0.52; 0.32	0.63
EMAS-SFQ erection ability							
[Na^+^]_G_ < 136 mmol/L	2.42 ± 1.10	− 0.05	− 0.12; 0.02	0.14	–	–	–
136 ≤ [Na^+^]_G_ ≤ 147 mmol/L	2.04 ± 1.03	− 0.02	− 0.03; − 0.01	0.02	0.03	− 0.04; 0.11	0.35
[Na^+^]_G_ > 147 mmol/L	2.26 ± 1.11	**0.15**	**0.04; 0.26**	** < 0.01**	**0.17**	**0.06; 0.28**	** < 0.01**
EMAS-SFQ erection worry							
[Na^+^]_G_ < 136 mmol/L	1.56 ± 0.96	0.02	− 0.05; 0.09	0.56	–	–	–
136 ≤ [Na^+^]_G_ ≤ 147 mmol/L	1.54 ± 0.91	− 0.01	− 0.03; 0.00	0.14	− 0.03	− 0.10; 0.04	0.40
[Na^+^]_G_ > 147 mmol/L	1.69 ± 1.09	**0.16**	**0.05; 0.27**	** < 0.01**	**0.18**	**0.07; 0.30**	** < 0.01**
EMAS-SFQ frequency worry							
[Na^+^]_G_ < 136 mmol/L	1.43 ± 0.84	0.01	− 0.07; 0.09	0.77	–	–	–
136 ≤ [Na^+^]_G_ ≤ 147 mmol/L	1.46 ± 0.83	− 0.01	− 0.01; 0.01	0.56	− 0.01	− 0.10; 0.07	0.72
[Na^+^]_G_ > 147 mmol/L	1.59 ± 0.95	**0.17**	**0.06; 0.28**	** < 0.01**	**0.19**	**0.08; 0.30**	** < 0.01**
EMAS-SFQ orgasm worry							
[Na^+^]_G_ < 136 mmol/L	1.63 ± 0.92	− 0.03	− 0.11; 0.05	0.51	–	–	–
136 ≤ [Na^+^]_G_ ≤ 147 mmol/L	1.50 ± 0.86	− 0.01	− 0.03; 0.00	0.11	0.02	− 0.07; 0.10	0.71
[Na^+^]_G_ > 147 mmol/L	1.74 ± 1.05	**0.17**	**0.06; 0.28**	** < 0.01**	**0.17**	**0.06; 0.28**	** < 0.01**
EMAS-SFQ morning erection worry							
[Na^+^]_G_ < 136 mmol/L	1.21 ± 0.64	− 0.01	− 0.06; 0.03	0.61	–	–	–
136 ≤ [Na^+^]_G_ ≤ 147 mmol/L	1.19 ± 0.61	− 0.01	− 0.01; 0.00	0.12	0.01	− 0.05; 0.05	0.88
[Na^+^]_G_ > 147 mmol/L	1.23 ± 0.63	**0.15**	**0.07; 0.23**	** < 0.01**	**0.16**	**0.08; 0.24**	** < 0.01**
PASE							
[Na^+^]_G_ < 136 mmol/L	207.87 ± 90.24	3.59	− 2.47; 9.64	0.24	–	–	–
136 ≤ [Na^+^]_G_ ≤ 147 mmol/L	195.69 ± 91.91	− 0.12	− 1.53; 1.29	0.87	− 3.79	− 10.26; 2.68	0.25
[Na^+^]_G_ > 147 mmol/L	160.04 ± 74.85	− 8.30	− 18.02; 1.42	0.09	− 8.61	− 18.56; 1.34	0.09

The B coefficient and the “B change” in table 2 are in bold when they are significant (as reported in table 2 significance was defined as p-value < 0.01 with Bonferroni adjustment)

The table reports the results from linear regression analyses using spline functions. The B coefficient refers to the change (slope) in the outcome variables for any unit change in the predictor variable ([Na^+^]_G_). The “B change” refers to the change in the slope from the preceding interval of [Na^+^]_G_ for any unit change in the predictor variable ([Na^+^]_G_). Significance was defined as p-value < 0.01 with Bonferroni adjustment

*SF36* the short form-36^18^, *EMAS-SFQ* European Male Aging Study sexual function questionnaire^20^, *PASE* Physical Activity Scale for the Elderly^19^, *SD* standard deviation

**Fig. 2:**
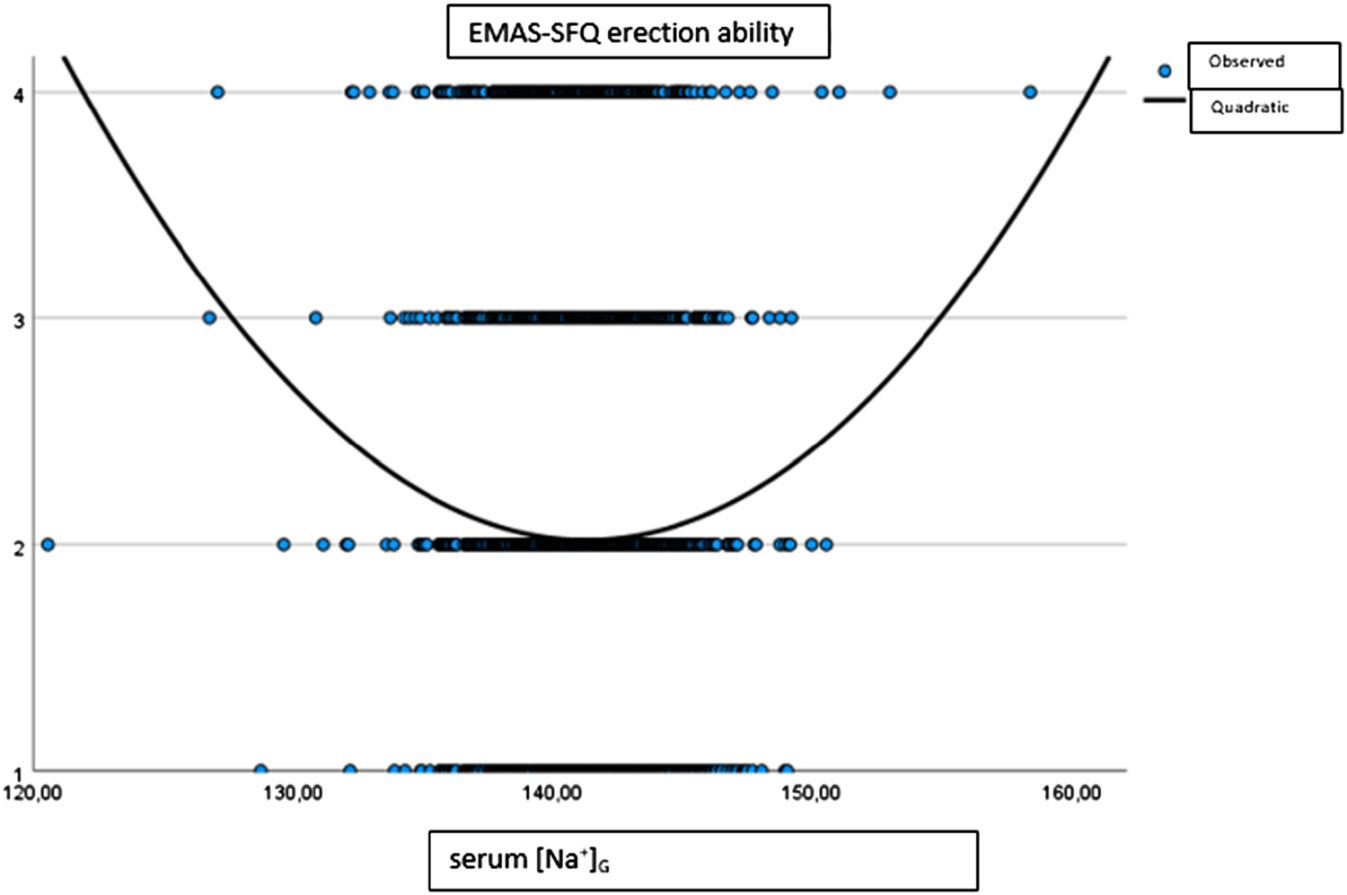
Relationship between serum [Na^+^]_G_ and erection ability

We repeated similar analyses for evaluating possible associations between serum [Na^+^]_G_ and sexual functioning. When considering the relationship between erectile dysfunction (EMAS-SFQ erection ability domain) and serum [Na^+^]_G_, we found a significant quadratic relationship (F = 9.00, p < 0.001) denoting that both for the highest and the lowest values of serum [Na^+^]_G_, a worsening of erection was detectable (Fig. 2).

We divided the subjects into the aforementioned groups according to serum [Na^+^]_G_ and, interestingly, when we performed a linear regression with spline functions, only adjusted for centre, we confirmed a significant worsening of erection in both patients with serum [Na^+^]_G_ > 147 mmol/L (B = 0.18 [0.04; 0.32], p = 0.01 for each unit increase in [Na^+^]_G_) and patients with serum [Na^+^]_G_ < 136 mmol/L (B = − 0.08 [− 0.15; − 0.01]; p = 0.04 for each unit increase in [Na^+^]_G_). When introducing age, morbidities, medications, BMI, and total testosterone as further covariates, only increases in serum [Na^+^]_G_ above 147 mmol/L were associated with a significantly worsened erectile function (Table 2). Accordingly, a significant change in the slope for the relationship between erectile function and serum [Na^+^]_G_ was observed in hypernatremic men as compared with normonatremic ones (Table 2). Since reporting concerns for sexual dysfunction is a pivotal point in sexual medicine, we analyzed worry about different domains of sexual function. Similarly, the relationship of serum [Na^+^]_G_ with worry about erection, sexual intercourse frequency, morning erection frequency, and orgasm was confirmed only for men with serum [Na^+^]_G_ > 147 mmol/L (Table 2).

## Discussion

This study shows a nonlinear relationship between serum [Na^+^]_G_ and mental, physical and sexual health in a large cohort of community-dwelling men from the European Male Ageing Study (EMAS) [15–17]. In fact, a worsening in mental health, physical activity and sexual symptoms was observed for values of serum [Na^+^]_G_ both in the highest and the lowest ends of the range, as pointed out by the U-shaped curve derived from the analyses on SF-36, PASE and EMAS-SFQ questionnaires. Interestingly, the present data show that in the hypo or hypernatremic categories, the symptomatology is dependent upon the severity of the alteration of serum [Na^+^]_G_ concentrations suggesting, therefore, that hypo or hypernatremia should be considered in light of the sodium value rather than absolute categories. These associations are in line with the most recent data from the literature, showing that hyponatremia, even when mild and chronic, is associated with alterations of psychological and physical domains [13, 26–28].

In fact, in collaboration with Suárez et al., we have recently demonstrated, in hospitalized patients, with confirmed euvolemic hyponatremia (< 130 mmol/L), that further decreases of serum sodium beyond mild hyponatremia are associated with an impairment in neurocognitive and motor performance as well as in mood disorders [13]. Consistently, the improvement of serum sodium after treatment resulted in significant benefits on the aforementioned functions [13]. Similar but less consistent findings have been published in recent years on patients with asymptomatic mild hyponatremia. Renneboog et al., demonstrated impaired gait stability, attention deficits and reduced alertness, with limitations resolved after restoration of normal sodium concentrations [26]. Conversely, in the INSIGHT trial, also in milder forms of hyponatremia, a cognitive performance deterioration was observed, although it was not a global impairment since cognitive speed domains declined with relatively preserved word and number recognition [27].

The effect of hyponatremia on mood disorders and the risk of depression has been also shown by the SALT placebo-controlled randomized trials, which reported a significant improvement in the mental health associated with tolvaptan treatment (a V2 vasopressin receptor antagonist), indicating a detrimental influence of hyponatremia on patients’ mood [28].

On the other hand, patients with hypernatremia may exhibit physical and neurological symptoms like muscle weakness [9], depression of sensorium, varying from moderate lethargy to coma [29], and seizures [30]. Signs and symptoms of hypernatremia depend on the entity and rapidity of the increase of serum sodium [7], but current literature lacks a structured analysis of the possible association between mild or chronic hypernatremia and psychological and physical domains.

In the literature, the relationship of serum sodium with sexual function has not been investigated. In our study, a worsening of erection for the highest and the lowest values of serum [Na^+^]_G_ was detected.

Interestingly, when we divided the subjects according to serum [Na^+^]_G_ (< 136, 136–147 and > 147 mmol/L) and we performed a linear regression with spline functions, we confirmed that erectile function declines for any further [Na^+^]_G_ changes. However, after introducing also age, morbidities, medications, BMI, and total testosterone, only increased serum [Na^+^]_G_ above 147 mmol/L was associated with a significantly worsened erectile function.

Interestingly, serum [Na^+^]_G_ concentrations above the threshold of 147 mmol/L were associated with worry about sexual function and, in particular, about erection, sexual intercourse frequency, morning erection frequency, and orgasm. This finding is particularly interesting. In fact, reporting concerns for sexual dysfunction is a pivotal point in sexual medicine because only impairments that cause distress identify an actual disorder and deserve further attention [31]. Therefore, it is noteworthy that hypernatremia, independently of other known risk factors, identifies men not only with erectile difficulties but also complaining about them. In an experimental mouse model of hyponatremia, we recently reported distinct testicular alterations mainly in the seminiferous tubules [32]. However, in that study, Leydig cell functions was not specifically investigated. In the present study, the association between hyponatremia and sexual dysfunction was not confirmed when possible confounders, including testosterone, were included in the model.

According to the aforementioned results, this study suggests that hyponatremia could represent an epiphenomenon of ill-health conditions, which may affect sexuality. Conversely, hypernatremia shows an association with sexual dysfunction, which is independent of factors commonly known to influence sexuality, such as ageing, chronic morbidities, medications or testosterone. The possible pathogenic mechanism to explain this independent association is not clear. In preclinical studies, increased plasma sodium concentration has been reported to have a detrimental effect on endothelial function. In particular, Oberleithner et al. demonstrated that increasing the concentration of sodium towards the upper limit of the reference range reduce nitric oxide release, which is a pivotal mediator for the erectile mechanism [33]. Moreover, elevated sodium can increase the production of von Willebrand factor by endothelial cells, another sign of endothelial dysfunction [34]. It has been proposed that reduced cell volume due to dehydration associated with hypernatremia could alter the cell function, thus providing another possible mechanism of endothelial dysfunction [35, 36].

Another possible vascular alteration in hypernatremia may be related to HDL cholesterol changes, since HDL remodeling with breakdown into small forms during hypernatremia has been reported [37] and this is an important point since a pro-atherogenic role of small HDL has been suggested [38]. Accordingly, hypernatremia has been reported as a significant predictor of adverse cardiac outcomes, including reduced left-ventricular ejection fraction, regional wall motion abnormalities of the left ventricle, elevated troponin, and pulmonary edema in patients with subarachnoid hemorrhage [39]. Even in the British general population without a history of cardiovascular disease, hypernatremic men showed a significantly higher risk of major cardiovascular events, in particular stroke and cardiovascular deaths [40], further emphasizing the possible vascular alterations mediated by hypernatremia, which could be related to erectile dysfunction.

Some limitations of this study should be recognized. The number of patients with high or low serum sodium concentrations is small, representing respectively 1.1% and 2.4% of the population. However, it should be considered that this prevalence is in line with other reports in older general population [39]. Moreover, lacking previous data on this topic and considering the exploratory nature of the present study, a formal sample size calculation is not feasible. Considering our sample size (36 men with serum [Na^+^]_G_ > 147 mmol/L), we estimate a power of 66.2% with an α error of 0.01 in detecting a significant association between serum [Na^+^]_G_ and erection ability. A power of 80% would be achieved with 46 men with serum [Na^+^]_G_ > 147 mmol/L. Therefore, we believe that our results are reliable, although needing further confirmations.

Our data cannot clarify the cause–effect relationship between serum [Na^+^]_G_ and impairment of mental health, physical activity and sexual function, which needs to be explored in longitudinal, epidemiological, and experimental human studies. The overall response rate for participation in the study was 41%, and it is possible that those who took part may have differed with respect to levels of physical, sexual and general health compared with those who declined to participate. This response bias may have led to an over or underestimation of the prevalence of sexual dysfunctions and comorbidities within the population sampled. Self-reported information in population surveys may be subject to errors of recall; however, any misclassification was likely to have been random, and the effect, if any, would be to reduce the reported associations toward the null rather than produce spurious associations.

## Conclusions

In line with the available literature, this study shows that middle-aged and older community-dwelling men with serum [Na^+^]_G_ in the highest or lowest ends of the range have a worsening in mental health and physical activity.

Furthermore, this is the first study to support a negative relationship between serum [Na^+^]_G_ and sexual functioning. A worsening of erection and, importantly, an increase in concern about sexual dysfunction including erectile dysfunction, sexual intercourse frequency, morning erection frequency, and orgasm was observed for the highest values of serum [Na^+^]_G_, independently of other relevant factors, including age, testosterone, chronic diseases, BMI, and medications. The possible reasons for these associations need further investigation in specifically designed studies. Therefore based on the current evidence, it is not possible to support serum [Na^+^]_G_ as a routine measurement for men with sexual dysfunction. Similarly, the correction of altered serum [Na^+^]_G_ cannot be considered as a treatment of sexual dysfunction. However, our findings indicate that serum [Na^+^]_G_ may play a role even in sexuality and its alteration should not be neglected and should be cautiously investigated [41].

## References

[1] Upadhyay A, Jaber BL, Madias NE (2006). Incidence and prevalence of hyponatremia. Am J Med.

[2] Hoorn EJ, Lindemans J, Zietse R (2006). Development of severe hyponatremia in hospitalized patients: treatment- related risk factors and inadequate management. Nephrol Dial Transplant: official publication of the European Dialysis and Transplant Association—European Renal Association.

[3] Sbardella E, Isidori AM, Arnaldi G (2018). Approach to hyponatremia according to the clinical setting: consensus statement from the Italian society of endocrinology (SIE), Italian society of nephrology (SIN), and Italian association of medical oncology (AIOM). J Endocrinol Investig.

[4] Adrogue HJ, Madias NE (2000). Hyponatremia. N Engl J Med.

[5] Ellison DH, Berl T (2007). Clinical practice. The syndrome of inappropriate antidiuresis. New Engl J Med.

[6] Adrogué HJ, Madias NE (2000). Hypernatremia. N Engl J Med.

[7] Snyder NA, Feigal DW, Arieff AI (1987). Hypernatremia in elderly patients: a heterogeneous, morbid, and iatrogenic entity. Ann Intern Med.

[8] Ross EJ, Christie SB (1969). Hypernatremia. Medicine (Baltimore).

[9] Hiromatsu K, Kobayashi T, Fujii N, Itoyama Y, Goto I, Murakami J (1994). Hypernatremic myopathy. J Neurol Sci.

[10] Pfennig CL, Slovis CM (2012). Sodium disorders in the emergency department: a review of hyponatremia and hypernatremia. Emerg Med Pract.

[11] Palevsky PM, Greenberg A (1998). Hypernatremia. Primer on kidney diseases.

[12] Corona G, Norello D, Parenti G, Sforza A, Maggi M, Peri A (2018). Hyponatremia, falls and bone fractures: a systematic review and meta-analysis. Clin Endocrinol.

[13] Suárez V, Norello D, Sen E (2020). Impairment of neurocognitive functioning, motor performance, and mood stability in hospitalized patients with euvolemic moderate and profound hyponatremia. Am J Med.

[14] Corona G, Giuliani C, Parenti G (2013). Moderate hyponatremia is associated with increased risk of mortality: evidence from a meta-analysis. PLoS One..

[15] Lee DM, O’Neill TW, Pye SR, EMAS study group (2009). The European male ageing study: design, methods and recruitment. Int J Androl.

[16] Corona G, Lee DM, Forti G, EMAS study group (2010). Age-related changes in general and sexual health in middle-aged and older men: results from the European male ageing study (EMAS). J Sex Med.

[17] Corona G, Wu FC, Forti G, EMAS study group (2012). Thyroid hormones and male sexual function. Int J Androl.

[18] Ware JE, Sherbourne CD (1992). The MOS 36-item short form health survey (SF-36): conceptual framework and item selection. Med Care.

[19] Washburn RA, Smith KW, Jette AM, Janney CA (1993). The physical activity scale for the elderly (PASE): development and evaluation. J Clin Epidemiol.

[20] O’Connor DB, Corona G, Forti G (2008). Assessment of sexual health in aging men in Europe: development and validation of the European male ageing study sexual function questionnaire. J Sex Med.

[21] O’Connor DB, Lee DM, Corona G, European Male Ageing Study Group (2011). The relationships between sex hormones and sexual function in middle-aged and older European men. J Clin Endocrinol Metab.

[22] Huhtaniemi IT, Tajar A, Lee DM, EMAS group (2012). Comparison of serum testosterone and estradiol measurements in 3174 European men using platform immunoassay and mass spectrometry; relevance for the diagnostics in aging men. Eur J Endocrinol.

[23] Alberti KG, Eckel RH, Grundy SM, International Diabetes Federation Task Force on Epidemiology and Prevention; Hational Heart, Lung, and Blood Institute; American Heart Association; World Heart Federation; International Atherosclerosis Society; International Association for the Study of Obesity (2009). Harmonizing the metabolic syndrome: a joint interim statement of the international diabetes federation task force on epidemiology and prevention; national heart, lung, and blood institute; American heart association; world heart federation; international atherosclerosis society; and international association for the study of obesity. Circulation.

[24] Katz MA (1973). Hyperglycemia-induced hyponatremia–calculation of expected serum sodium depression. N Engl J Med.

[25] Hillier TA, Abbott RD, Barrett EJ (1999). Hyponatremia: evaluating the correction factor for hyperglycemia. Am J Med.

[26] Renneboog B, Musch W, Vandemergel X, Manto MU, Decaux G (2006). Mild chronic hyponatremia is associated with falls, unsteadiness, and attention deficits. Am J Med.

[27] Verbalis JG, Ellison H, Hobart M, Krasa H, Ouyang J, Czerwiec FS, Investigation of the Neurocognitive Impact of Sodium Improvement in Geriatric Hyponatremia: Efficacy and Safety of Tolvaptan (INSIGHT) Investigators (2016). Tolvaptan and neurocognitive function in mild to moderate chronic hyponatremia: a randomized trial (INSIGHT). Am J Kidney Dis: the official Journal of the National Kidney Foundation.

[28] Schrier RW, Gross P, Gheorghiade M, SALT Investigators (2006). Tolvaptan, a selective oral vasopressin V2-receptor antagonist, for hyponatremia. New Engl J Med.

[29] Arieff AI, Guisado R (1976). Effects on the central nervous system of hypernatremic and hyponatremic states. Kidney Int.

[30] Finberg L, Harrison HE (1955). Hypernatremia in infants; an evaluation of the clinical and biochemical findings accompanying this state. Pediatrics.

[31] McCabe MP, Sharlip ID, Atalla E (2016). Definitions of sexual dysfunctions in women and men: a consensus statement from the fourth international consultation on sexual medicine 2015. J Sex Med.

[32] Marroncini G, Anceschi C, Naldi L (2023). Hyponatremia-related liver steatofibrosis and impaired spermatogenesis: evidence from a mouse model of the syndrome of inappropriate antidiuresis. J Endocrinol Invest.

[33] Oberleithner H, Riethmüller C, Schillers H (2007). Plasma sodium stiffens vascular endothelium and reduces nitric oxide release. Proc Natl Acad Sci USA.

[34] Dmitrieva NI, Burg MB (2014). Secretion of von Willebrand factor by endothelial cells links sodium to hypercoagulability and thrombosis. Proc Natl Acad Sci USA.

[35] Haussinger D (1996). The role of cellular hydration in the regulation of cell function. Biochem J.

[36] Haussinger D, Roth E, Lang F, Gerok W (1993). Cellular hydration state: an important determinant of protein catabolism in health and disease. Lancet.

[37] Andreeva AM, Martemyanov V, Vasiliev Ilya AS, Lamash N, Garina DV, Pavlov D (2022). Goldfish as a model for studying the effect of hypernatremia on blood plasma lipoproteins. Bratisl Lek Listy.

[38] Hluchanova A, Kollar B, Klobucnikova K (2023). Lipoprotein subfractions associated with endothelial function in previously healthy subjects with newly diagnosed sleep apnea-a pilot study. Life (Basel, Switzerland).

[39] Fisher LA, Ko N, Miss J (2006). Hypernatremia predicts adverse cardiovascular and neurological outcomes after SAH. Neurocrital Care.

[40] Wannamethee SG, Shaper AG, Lennon L, Papacosta O, Whincup P (2016). Mild hyponatremia, hypernatremia and incident cardiovascular disease and mortality in older men: a population-based cohort study. Nutr Metab Cardiovasc Dis.

[41] Arnaldi G, Arvat E, Berton AM (2023). Endocrinologists at work: management of hyponatremia in clinical practice. J Endocrinol Invest.

